# Functional analysis of monoclonal antibodies against the *Plasmodium falciparum* PfEMP1-VarO adhesin

**DOI:** 10.1186/s12936-015-1016-5

**Published:** 2016-01-15

**Authors:** Micheline Guillotte, Farida Nato, Alexandre Juillerat, Audrey Hessel, Françoise Marchand, Anita Lewit-Bentley, Graham A. Bentley, Inès Vigan-Womas, Odile Mercereau-Puijalon

**Affiliations:** Institut Pasteur, Unité d’Immunologie Moléculaire des Parasites, 25-28 rue du Dr ROUX, 75015 Paris, France; URA CNRS 2581, 25-28 rue du Dr ROUX, 75015 Paris, France; Institut Pasteur, Plate-forme de Production de Protéines recombinantes et d’Anticorps (PF5), 25-28 rue du Dr ROUX, 75015 Paris, France; Institut Pasteur, Unité d’Immunologie Structurale, 25-28 rue du Dr ROUX, 75015 Paris, France; CNRS URA 2185, 25-28 rue du Dr ROUX, 75015 Paris, France; Institut Pasteur de Madagascar, Unité d’Immunologie des Maladies Infectieuses, BP 1274, Antananarivo 101, Madagascar

**Keywords:** Malaria, Rosetting, PfEMP1 adhesin, Monoclonal antibodies (mAbs), Rosette-disrupting antibodies, Epitopes, Antigenicity

## Abstract

**Background:**

Rosetting, namely the capacity of the *Plasmodium falciparum*-infected red blood cells to bind uninfected RBCs, is commonly observed in African children with severe malaria. Rosetting results from specific interactions between a subset of variant *P. falciparum* erythrocyte membrane protein 1 (PfEMP1) adhesins encoded by *var* genes, serum components and RBC receptors. Rosette formation is a redundant phenotype, as there exists more than one *var* gene encoding a rosette-mediating PfEMP1 in each genome and hence a diverse array of underlying interactions. Moreover, field diversity creates a large panel of rosetting-associated serotypes and studies with human immune sera indicate that surface-reacting antibodies are essentially variant-specific. To gain better insight into the interactions involved in rosetting and map surface epitopes, a panel of monoclonal antibodies (mAbs) was investigated.

**Methods:**

Monoclonal antibodies were isolated from mice immunized with PfEMP1-VarO recombinant domains. They were characterized using ELISA and reactivity with the native PfEMP1-VarO adhesin on immunoblots of reduced and unreduced extracts, as well as SDS-extracts of Palo Alto 89F5 VarO schizonts. Functionality was assessed using inhibition of Palo Alto 89F5 VarO rosette formation and disruption of Palo Alto 89F5 VarO rosettes. Competition ELISAs were performed with biotinylated antibodies against DBL1 to identify reactivity groups. Specificity of mAbs reacting with the DBL1 adhesion domain was explored using recombinant proteins carrying mutations abolishing RBC binding or binding to heparin, a potent inhibitor of rosette formation.

**Results:**

Domain-specific, surface-reacting mAbs were obtained for four individual domains (DBL1, CIDR1, DBL2, DBL4). Monoclonal antibodies reacting with DBL1 potently inhibited the formation of rosettes and disrupted Palo Alto 89F5 VarO rosettes. Most surface-reactive mAbs and all mAbs interfering with rosetting reacted on parasite immunoblots with disulfide bond-dependent PfEMP1 epitopes. Based on competition ELISA and binding to mutant DBL1 domains, two distinct binding sites for rosette-disrupting mAbs were identified in close proximity to the RBC-binding site.

**Conclusions:**

Rosette-inhibitory antibodies bind to conformation-dependent epitopes located close to the RBC-binding site and distant from the heparin-binding site. These results provide novel clues for a rational intervention strategy that targets rosetting.

**Electronic supplementary material:**

The online version of this article (doi:10.1186/s12936-015-1016-5) contains supplementary material, which is available to authorized users.

## Background

Sequestration of mature *Plasmodium falciparum* intra-erythrocytic stages in the microvasculature is a major contributor to falciparum pathogenesis [1, 2]. The best-characterized parasite factor implicated in cytoadherence is the family of *P*. *falciparum* erythrocyte membrane protein 1 (PfEMP1) variant adhesins encoded by the approximately 60-member *var* gene family [3]. PfEMP1 molecules comprise a large surface-exposed N-terminal region containing a suite of modules called Duffy-Binding Like (DBL) domains and Cysteine-rich Inter-Domain Regions (CIDR), a single transmembrane segment and a cytoplasmic C-terminal domain. DBL and CIDR domains are highly variable within the PfEMP1 family but they can be assigned to a limited number of classes according to distinct sequence signatures [4–6]. The variability in sequence and domain organization in PfEMP1 variants [6] provides the parasite with the capacity to bind to an array of host receptors and to evade host immunity [3].

The capacity of infected red blood cells (iRBCs) to cyto-adhere to uninfected RBC, i.e., rosetting, has been associated with severe malaria in African children, with higher frequency of rosette-forming parasites and larger rosettes than in uncomplicated malaria [7–11]. Rosetting is also associated with an elevated infecting parasite biomass [10] and an increased multiplication rate in a non-human primate model [12]. Rosetting involves specific interactions between a subset of PfEMP1 adhesins [5, 6, 13–15], serum factors [15–22] and a variety of RBC receptors [20, 23–26]. Using vaccination or soluble inhibitors to target rosetting is thus an attractive strategy against severe malaria pathology.

To better understand critical molecular interactions and immunologic determinants implicated in rosetting, experimental models are needed. The Palo Alto VarO, a clonal rosetting line infectious for the *Saimiri sciureus* monkey [12], has been developed as a monovariant culture, in which a large majority (90–95 %) of the iRBCs express the Palo Alto varO gene [13]. The PfEMP1-VarO extracellular region has five DBL domains (DBL_1–5_) and one CIDR domain. All six domains, as well as the double DBL1-CIDR Head domain, have been produced as recombinant proteins [13, 20, 27, 28]. RBC binding has been mapped to DBL1α and the ABO blood group determinants have been identified as the erythrocyte receptor [20]. This model was used to explore the immune response of humans living in endemic areas showing elevated seroprevalence in Senegalese [13] and Beninese settings [29]. Two important features emerged from these studies, namely that the surface-reacting antibodies acquired by humans exposed to malaria were variant-specific [30] and that there were no rosette-disrupting antibodies in children [29].

Previous work has shown that DBL1, CIDR1, DBL2, DBL4 and the Head PfEMP1-VarO domains elicited antibodies reacting with the Palo Alto VarO iRBC surface. The work reported here aims to gain insight into the surface epitopes of PfEMP1-VarO using monoclonal antibodies (mAbs) isolated from mice immunized with these recombinant domains. The mAbs were characterized with respect to reactivity with the iRBC membrane-anchored PfEMP1-VarO by surface immunofluorescence and immunoblots of SDS-extracts of Palo Alto VarO iRBCs. Their functionality was assessed using rosette disruption and inhibition of rosette formation assays. The reactivity of surface-reacting mAbs specific for DBL1 was analysed using a panel of mutant domains, highlighting the existence of two distinct binding sites of potent rosette-disrupting mAbs. These results provide novel clues for the design of anti-rosetting strategies.

## Methods

### Parasites and rosetting assays

89F5 Palo Alto VarO parasites were cultivated as described in O^+^ RBC in RPMI 1640 medium containing l-glutamine and 25 mM HEPES (called hereafter RPMI) and supplemented with 10 % AB^+^ human serum [13]. Rosetting parasites were enriched once a week on ice-cold Ficoll and rosetting rate was kept >90 % by panning on a specific monoclonal antibody as described [13, 30]. Procedures and reagents for monovariant culture, and rosette purification have been published in detail [31].

For inhibition of rosette formation, monovariant VarO cultures were treated with 0.3 M Alanine (in 10 mM Hepes) to eliminate all mature stages. The cultures were then adjusted to 5 % parasitemia, 2 % hematocrit in RPMI, 10 % human AB^+^ serum and 100 µL aliquots (ring stages) were incubated in 96-well plates at 37 ℃ for 24 h with serial dilutions of monoclonal IgG or control reagents (diluted in RPMI, 10 % human AB^+^ serum). After this 24-h cultivation, parasite nuclei were labelled with 10 µg mL^−1^ Hoechst 33342 (Molecular Probes^®^ Thermo Fisher Scientific) for 10 min at 37 ℃ and an aliquot of the culture was examined using a fluorescence microscope. The number of iRBCs engaged in rosettes were scored by examining 200 iRBCs. The rosetting rate was calculated as the percent of mature stage iRBCs engaged in rosettes. Details are described in [31].

The rosette disruption assay has been described in detail elsewhere [32, 33]. Briefly, monovariant Palo Alto 89F5 VarO rosette preparations, adjusted to 5 % parasitemia, 5 % hematocrit in RPMI, 10 % human AB^+^ serum were incubated 30 min at 37 ℃ with serial dilutions of monoclonal IgG or control reagents (diluted in RPMI, 10 % human AB^+^ serum). At the end of incubation, mature stages engaged in rosettes or free of bound RBCs were numerated by microscopy (for details see [31, 32]). The rosetting rate was then evaluated as above.

### Production of recombinant VarO domains

The recombinant domains used to immunize mice and isolate mAbs are shown in Table 1, which indicates their type alongside some sequence features. Cloning of the re-codoned DBL3 and DBL5 domains (which did not elicit surface-reacting antisera) has been described previously [20]. The various DBL1 mutants have been described in [20, 28]; the Genbank accession number of the wild type protein sequence is EU908205. Recombinant proteins were stored in aliquots at −80 ℃ and thawed before use. This, however, caused internal cleavage in some proteins.

**Table 1:** List of recombinant PfEMP1-VarO domains, sequence features and expression systems used to immunise mice

VarO domain	Amino acid position in the complete protein sequence	Sequence features (number of potential N-glycosylation sites mutated S/T to A)	Expression system	Name used	References
DBL1α1.6	1–487	Recodoned (7)	Baculovirus/insect cells	bDBL1	[13]
			*E. coli*/pMAL-c2X	eDBL1	[27]
CIDRγ6	399–835	Recodoned (1)	Baculovirus/insect cells	bCIDR	[33]
	508–787	Recodoned (1)	*P. pastoris*/pPICZαA	pCIDR	[20]
Head (DBL1α1.6–CIDRγ6)	2–716	Recodoned (8)	*E. coli*/pMAL-c2X	eHead	[20]
DBL2β7	821–1242	Recodoned (2)	Baculovirus/insect cells	bDBL2	[33]
DBL4ε5	1608–2014	Recodoned (3)	*E. coli*/pMAL-c2X	eDBL4	[20]

### Production of monoclonal antibodies (mAbs)

MAbs were obtained from OF1 mice immunized with bDBL1, eHead, pCIDR1, bDBL2, and eDBL4 (10 µg/dose injected subcutaneously at 3-week intervals, mixed with complete Freund’s adjuvant for the first dose and incomplete for the boosters) [33]. Mice with high specific IgG titres against the immunogen received an intraperitoneal boost immunization 4 days before being sacrificed for splenic B cell fusion, which was performed as described [34]. For bDBL1, culture supernatants were screened for VarO iRBC surface reactivity by flow cytometry (hybridomas D15-50, D15-68, E20-76). For pCIDR, eHead, bDBL2, and eDBL4, culture supernatants were first screened by ELISA on the recombinant protein and positive culture supernatants were then tested by surface-IFA/flow cytometry against VarO iRBCs. Positive hybridomas were cloned by limiting dilution and screened for ELISA and VarO iRBC surface reactivity. Hybridomas BD20E4 and BDEE10 were custom-made by RD Biotech (Besançon, France), who performed the screening by ELISA on the recombinant protein. Clones were then selected on the basis of VarO iRBC surface reactivity by flow cytometry in the authors’ laboratory. Monoclonal IgG were precipitated with 50 % ammonium sulfate from ascitic fluid, centrifuged and dialyzed against PBS. Monoclonal Ig class and sub-class were determined by ELISA using the isotrip mouse mAb isotyping kit (Roche). Monoclonal IgG were purified using the Melon™ gel monoclonal IgG purification kit (Thermo Fisher Scientific) as recommended by the manufacturers. D15-50, E20-76 and BD20E4 IgG were biotinylated using EZ-link sulfo-NHS-biotin (Thermo Fisher Scientific) and centrifuged through Zeba™ spin desalting columns (Thermo Fisher Scientific) according to the supplier’s recommendations. The concentration of the biotinylated antibodies was evaluated using the Pierce BCA protein assay kit (Thermo Fisher Scientific).

### Ethics

The study was carried out in strict accordance with the recommendations in the guide for the care and use of laboratory animals of the Institut Pasteur and complied with the French [35] and European Union guidelines for the handling of laboratory animals [36]. Animal care and handling was approved by the Ministère de l’Agriculture et de la Pêche (Ref 107503056792, issued to OMP) and the protocols and procedures approved by the Direction Départementale des Services Vétérinaires du Préfet de Police de Paris (clearance number C75-273 issued to OMP). All animal experiments were planned and executed in order to minimize animal suffering.

### ELISA assays

ELISA plates (Nunc maxisorp) were coated with 100 μL/well of a solution of 0.2 μg mL^−1^ recombinant protein in PBS, overnight at 4 ℃. Non-specific absorption was blocked with 200 μL blocking buffer (PBS, 5 % w/v non-fat milk) for 1 h at 37 ℃, 100 μL serial dilutions of sera (in PBS, 2.5 % non-fat milk, 0.05 % Tween-20) were incubated 1 h at 37 ℃. Plates were then washed three times with washing buffer (PBS, 0.1 % Tween-20) and incubated for 1 h at 37 ℃ with horseradish peroxidase-conjugated goat anti-mouse IgG (H + L) (Promega) diluted at 1/3000 or anti-rabbit IgG (Promega) diluted 1/3000 in washing buffer. Plates were washed three times before incubation for 5 min at RT with 100 μL TMB/H_2_O_2_ substrate (KPL). The enzymatic reaction was stopped by addition of 100 μL 1 M H_3_PO_4_. Absorbance was measured at 450–655 nm. Each serum dilution was tested in duplicate.

For competition ELISA with biotinylated IgG, plates coated with 1 μg mL^−1^ bDBL1 were incubated with unlabelled monoclonal D15-50, D15-68, E20-76, BD20E4, BDEE10, M21-17 and M21-30 IgG at saturating concentration (50 μg mL^−1^) for 2 h at 37 ℃. After four washes with PBS, 0.1 % Tween-20, biotinylated D15-50, E20-76 or BD20E4 IgG were added to individual wells at a concentration previously determined to generate a signal of approximately 1 OD after incubation at 4 ℃ for 20 min. After this 20-min incubation and four successive washes with washing buffer, streptavidin-conjugated horseradish peroxidase (Thermo Fisher Scientific) diluted 1/1000 was added to each well and incubated for 60 min at 37 ℃ and after four successive washes with washing buffer, the bound enzyme was revealed as above.

### Immunoblots

Immunoblots of non-reduced recombinant proteins were prepared as described [33]. Briefly, proteins were fractionated on 4–12 % SDS-PAGE gradient and transferred to a nitrocellulose membrane (Amersham). The membranes were blocked with PBS, 5 % non-fat milk for 1 h at room temperature and probed with diluted sera in PBS, 2.5 % non fat milk, 0.05 % Tween-20. After three washes with PBS, 0.1 % Tween-20, the blot was incubated with anti-mouse IgG (H + L) conjugated to alkaline phosphatase (Promega) diluted 1/10,000 in PBS, 2.5 % non fat milk, 0.05 % Tween-20 for 1 h at room temperature, washed and binding was revealed using Western Blue^®^ stabilized substrate (Promega) as recommended by the supplier.

Immunoblots of Palo Alto 89F5 VarO crude extracts were prepared as described [37] from rosettes selected on ice-cold Ficoll and enriched on magnetic columns (VarioMacs and CS MACS separation columns, Miltenyi Biotec) to obtain a preparation containing >95 % iRBCs at the mature stage [31]. For SDS-extracts preparations, the highly enriched parasite preparation was first resuspended in 20 volumes of Triton buffer (PBS, 1 % Triton X-100, 2 mM EGTA, complete EDTA-free protease cocktail inhibitors from Roche diagnostics) for 30 min at 4 ℃ and centrifuged at 16,100*g* at 4 ℃ for 30 min. The pellet was then washed twice with Triton buffer and insoluble proteins were extracted with PBS, 2 % sodium dodecyl sulfate (SDS) supplemented with protease inhibitors for 30 min at RT under a rotation movement and centrifuged at 16,100*g* for 30 min at 4 ℃. The supernatant was collected and diluted tenfold with PBS, 10 % Triton. The SDS-soluble protein extract was kept at −80 ℃ until use. Immunoblots prepared from reduced or non-reduced SDS-extracts were processed as above.

### Surface immunofluorescence assay

Antibodies (IgG) reacting with the PfEMP1-VarO protein displayed on iRBC surface were monitored by indirect immunofluorescence using flow cytometry or fluorescence microscopy as described [33]. Immunofluorescence staining was analysed as described [33].

## Results

### Isolation and characterization of monoclonal antibodies

Hybridomas were generated from mice immunized with bDBL1, pCIDR, bDBL2, eDBL4 or the eHead domain (Table 1). Initial screening for surface reactivity identified mAbs E20-76, D15-50 and D15-68 from mice immunized with bDBL1. Screening of subsequent fusions was done stepwise, first by ELISA on the immunizing antigen and then by VarO iRBC surface reactivity. Overall seven anti-bDBL1, two anti-pCIDR, two anti-bDBL2 and one anti-DBL4 mAbs were studied in detail. All anti-bDBL1 mAbs and the anti-pCIDR mAbs were IgG1, while mAb G8-49 was classified as IgG2b and mAbs B12-15, B12-42 and D18-94 were IgG2a (Table 2).

**Table 2:** ELISA titres, parasite immunoblot reactivity, surface reactivity and functional characteritics of anti-PfEMP1 VarO monoclonal IgGs

mAb	Immunising antigen	Isotype	ELISA		Palo Alto 89F5 VarO iRBC								
					Immunoblot				Surface IFA		Rosette inhibition^b^	Rosette disruption^b^	
					SDS-extract		Total extract						
			50 % titre^a^	Endpoint titre^a^	Reduced	Non-reduced	Reduced	Non-reduced	Endpoint titre^a^	MFI max	100 %	100 %	50 %
D15-50	bDBL1	IgG1	10	0.01	Neg	Pos	Neg	Pos	1.6	700	1	100	5
D15-68	bDBL1	IgG1	10	0.03	Neg	Pos	Neg	Pos	10	400	>10	≫100	100
E20-76	bDBL1	IgG1	10	0.015	Neg	Pos	Neg	Pos	3.2	300	5	≫100	20
M21-17	bDBL1	IgG1	200	1.2	Neg	Neg	Neg	Pos	Neg	Neg	NA	NA	NA
M21-30	bDBL1	IgG1	330	1.2	Neg	Neg	Neg	Pos	Neg	Neg	NA	NA	NA
BDEE10	bDBL1	IgG1	10	0.03	ND	ND	Pos	Pos	16	200	>50	>100	40
BD20E4	bDBL1	IgG1	10	0.03	Neg	Pos	Neg	Pos	0.6	700	1	10	0.7
N6-37	pCIDR	IgG1	2	0.015	ND	ND	Neg	Pos	10	200	≫50	Nil	Nil
G8-49	eHead	IgG2b	20	0.1	ND	ND	Pos	Pos	2000	100	Nil	Nil	Nil
B12-15	bDBL2	IgG2a	1.4	0.005	Neg	Pos	Neg	Pos	3.2	400	Nil	Nil	Nil
B12-42	bDBL2	IgG2a	1.4	0.01	Neg	Pos	Neg	Pos	3.2	400	Nil	Nil	Nil
D18-94	eDBL4	IgG2a	15	0.01	ND	ND	Pos	Pos	10	350	Nil	Nil	Nil
Rabbit^c^	bDBL1		78	<4.8	Pos	Pos	Pos	Pos	5	650	25	>100	35

*ND* not determined; *NA* not applicable; *nil* no inhibition or disruption at the highest concentration tested 50 and 100 µg ml^−1^ respectively

^a^Concentration of monoclonal IgG (ng mL^−1^)

^b^Concentration of mAb (µg mL^−1^)

^c^Ref [33]

**Fig. 1:**
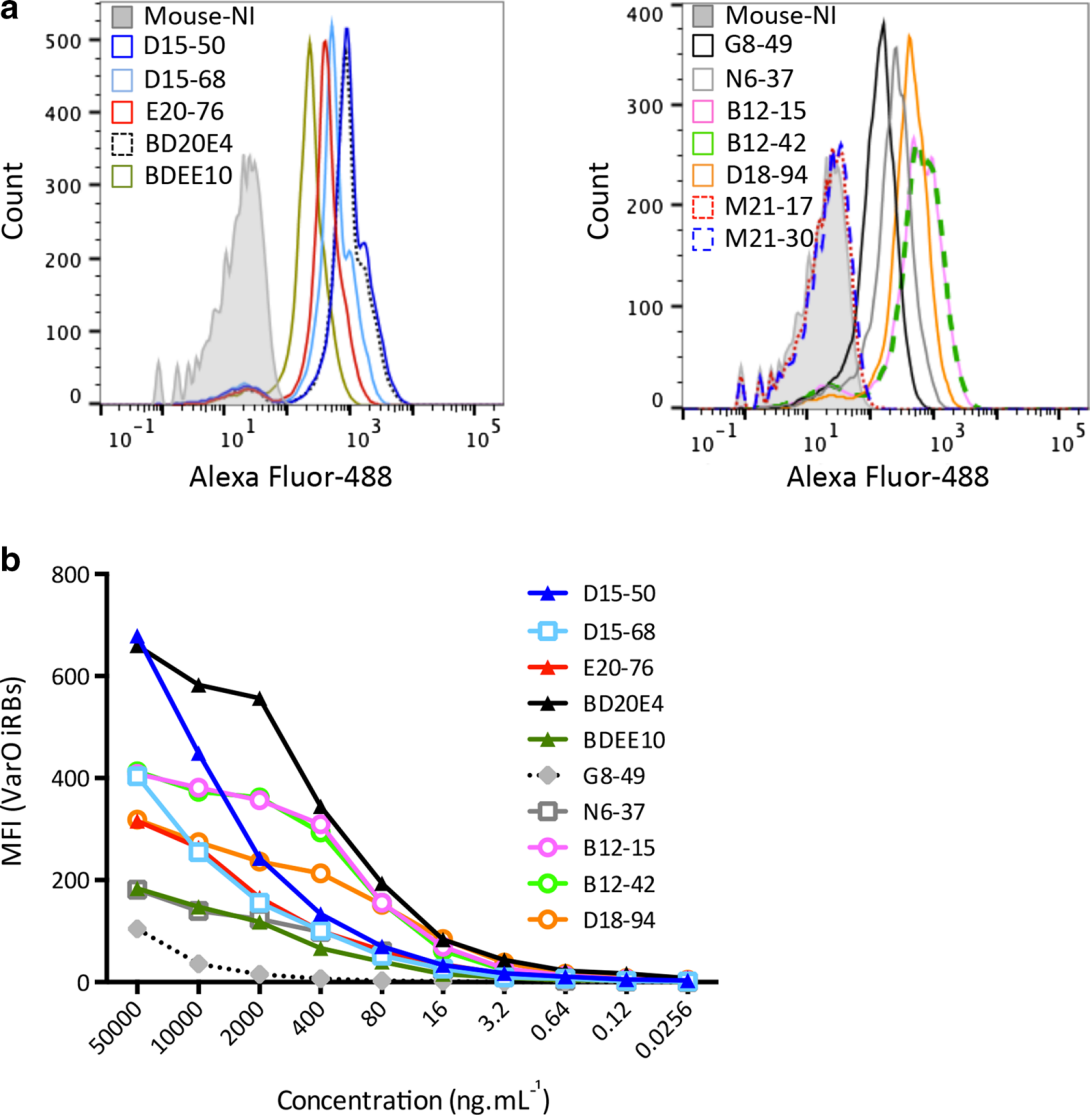
Surface reactivity of anti-PfEMP1-VarO mAbs assessed by flow cytometry. **a** Mean fluorescence intensity (MFI) of each anti-PfEMP1 VarO mAb reacting with the PfEMP1-VarO protein displayed on the surface of monovariant Palo Alto 89F5 VarO rosette-forming iRBC (trophozoite stage). Each mAb was used at 10 μg mL^−1^ and surface reactivity was monitored by indirect immunofluorescence using a goat anti-mouse IgG Alexa Fluor 488-conjugated antibody. The shaded histograms show labelling with a pool of mouse non-immune sera. **b** Titration curves of surface reactivity for individual mAbs, which are *colour-coded* as indicated

MAbs M21-17 and M21-30 reacted with bDBL1 and eDBL1 by ELISA but not with the Palo Alto 89F5 VarO iRBC surface (Fig. 1a). All other mAbs studied were surface-reactive as well as reacting by ELISA on the cognate domain. Figure 1a shows profiles of surface reactivity assessed by flow cytometry and Fig. 1b the titration curves of individual mAbs by flow cytometry.

### Domain specificity

**Fig. 2:**
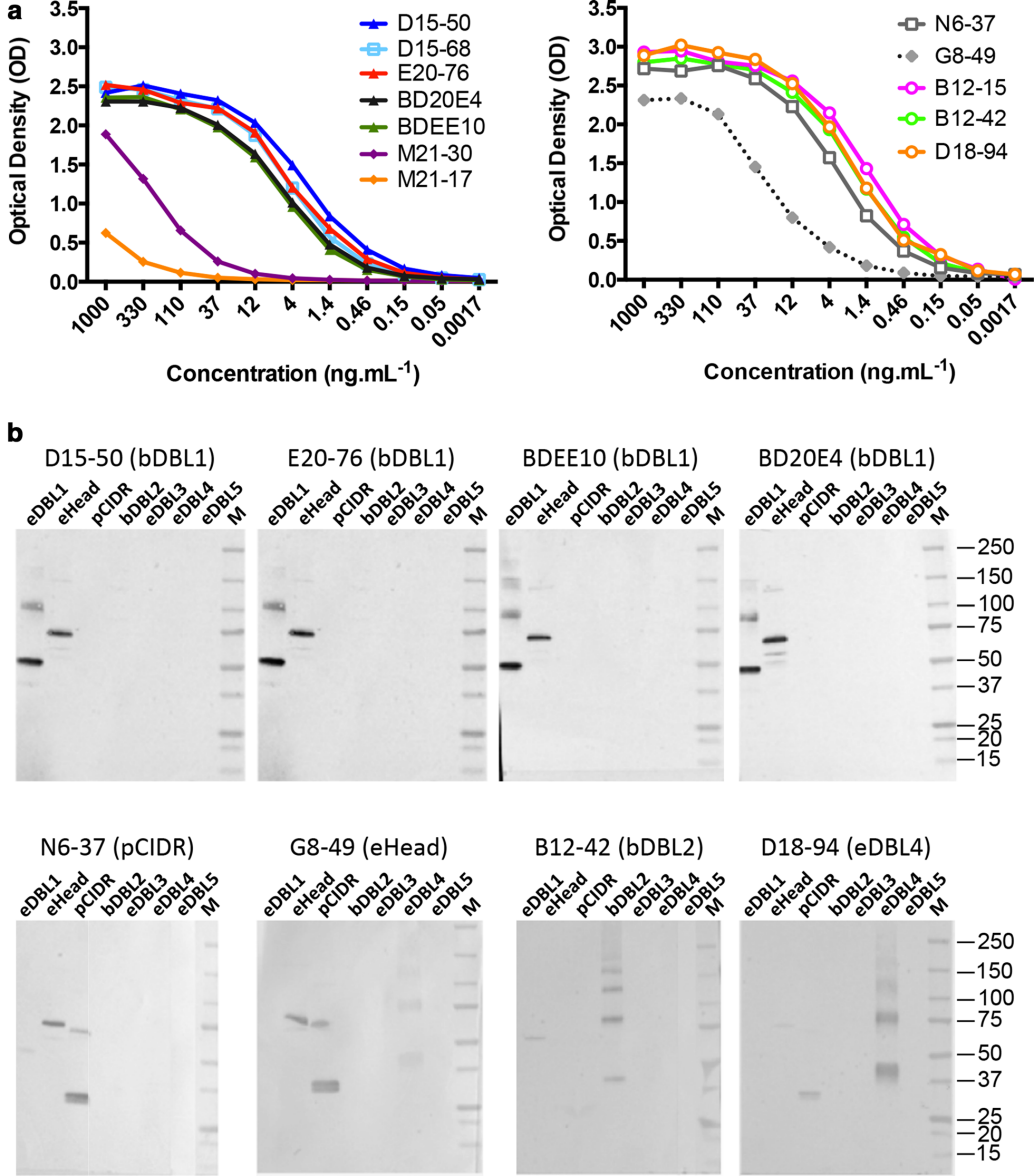
Reactivity of anti-PfEMP1-VarO mAbs on cognate and other domains assessed by ELISA and immunoblot. **a** ELISA titration curves of mAbs on the cognate domain. ELISAs were carried out as described in the “Methods” section. The antigen used to coat the plate was as follows: eDBL1 for mAbs D15-50, D15-68, E20-76, BD20E4, BDEE10, pCIDR for N6-37 and G8-49, eDBL2 for B12-15 and B12-42, eDBL4 for D18-94. Each dilution was tested in duplicate. **b** Specificity of the mAbs raised to PfEMP1-VarO-derived recombinant domains assessed by immunoblot on the cognate and other PfEMP1-VarO-derived recombinant domains. Recombinant domains (50 ng each) were separated on 4–12 % SDS gels under non-reducing conditions and immunoblotted. Eight immunoblots were prepared in parallel and loaded with eDBL1, eHead, pCIDR, bDBL2, eDBL3, eDBL4, and eDBL5 as indicated. Blots were incubated with individual mAbs (20 μg mL^−1^) as indicated

All mAbs reacted by ELISA with the cognate domain irrespective of the expression system (Fig. 2a). Immunoblotting showed that the anti-DBL1 mAbs D15-50, E20-76 and BDEE10 reacted with the cognate recombinant antigen and not with the other domains (Fig. 2b). There was very faint cross-reactivity of the anti-CIDR mAb N6-37 with eDBL1; this was not observed with mAb G8-49 (raised to eHead), which presented faint cross-reactivity to eDBL4. MAb B12-42 (raised to bDBL2) showed some faint cross-reactivity with eDBL1 while mAb D18-94 (raised to eDBL4) cross-reacted with pCIDR.

### Reactivity with the PfEMP1 protein on parasite immunoblots

**Fig. 3:**
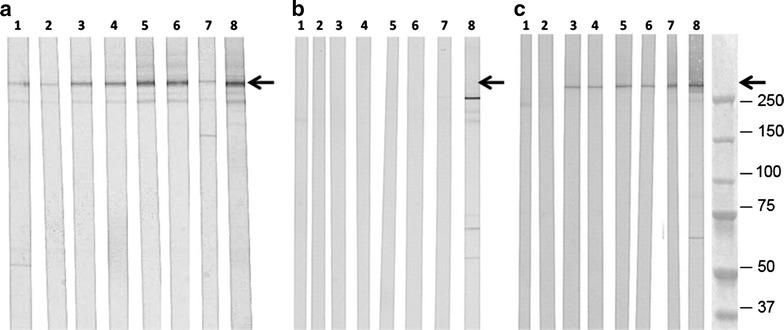
Reactivity of mAbs with the native parasite PfEMP1-VarO protein on immunoblot of Palo Alto 89F5 VarO extracts. Non-reduced total schizont extract (**a**); reduced SDS extract of schizonts (**b**); non-reduced SDS extract of schizonts (**c**). Strips from each blot were reacted with 50 μg mL^−1^ mAbs as follows: mAb M21-17 (*lane 1*), mAb M21-30 (*lane 2*), mAb E20-48 (*lane 3*), mAb E20-76 (*lane 4*), mAb D15-50 (*lane 5*), mAb D15-68 (*lane 6*), mAb N6-37 (*lane 7*), and mAb B12-15 (*lane 8*). The *arrow* indicates PfEMP1-VarO

All mAbs reacted on immunoblots of non-reduced total extracts of Palo Alto 89F5 VarO iRBCs but only mAbs BDEE10, G8-49 and D18-94 reacted with the reduced extract (Fig. 3a shows the pattern of reactivity of seven mAbs with the non-reduced total extract, see also Table 2). The surface-displayed PfEMP1 antigen is a type I membrane protein that can be solubilized in 0.5 % SDS. Probing of immunoblots of SDS-extracts showed that mAbs M21-17 and M21-30, which reacted with PfEMP1-VarO on immunoblots of non-reduced total parasite extracts (Fig. 3a, lanes 1 and 2, respectively), failed to react on the SDS-solubilized, membrane-anchored PfEMP1-VarO (Fig. 3b, c, lanes 1 and 2, respectively). This suggests that these mAbs recognize an epitope that is not present on the membrane-anchored PfEMP1-VarO. All surface-reacting mAbs tested reacted with the SDS-extracted PfEMP1-VarO and this reaction was abolished upon reduction of the parasite proteins (Fig. 3b, c). Table 2 summarizes ELISA titres, parasite immunoblot reactivity and surface reactivity of the various mAbs.

### Competition ELISA

Competition ELISAs using individual biotinylated anti-bDBL1 mAbs were used to classify the mAbs. Biotinylation reduced the ELISA titre of each mAb on the cognate antigen by approximately threefold as indicated by the shift of the antigen-dose dependent titration curves (see Additional file 1: Figure S1**)** but as its impact was similar for the three mAbs, competition ELISAs were carried out. This distributed the mAbs into three non-competing sets: (i) D15-50 and E20-76; (ii) BD20E4 and BDEE10; and (iii) M21-30 and M21-17, although each biotinylated mAb competed best with its non-biotinylated cognate mAb (see Additional file 2: Table S1). As M21-30 and M21-17 did not react with the iRBC surface, they were not studied further with regard to functionality.

### Functional characterization: inhibition of rosette formation and rosette disruption

To assess the capacity of the mAbs to interfere with VarO rosetting, two functional assays were carried out with monovariant Palo Alto 89F5 VarO cultures, i.e., cultures in which >90 % of the mature stages formed rosettes that reacted with any of the anti-VarO mAbs. Data are summarized in Table 2.

**Fig. 4:**
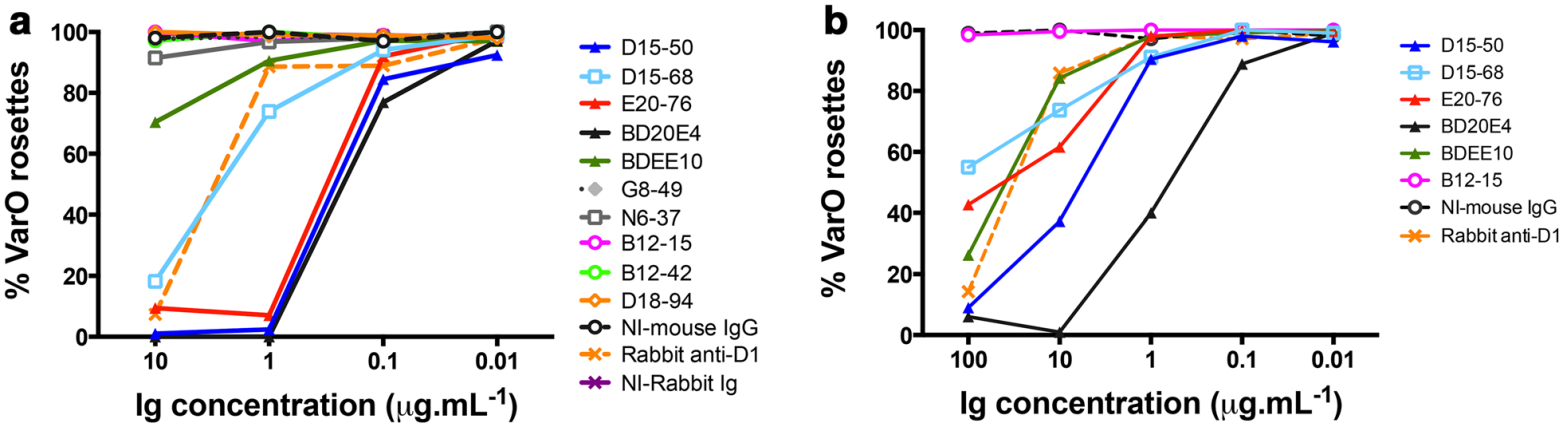
Titration of the capacity to inhibit formation of Palo Alto 89F5 VarO rosettes (**a**) or disrupt Palo Alto 89F5 VarO rosettes (**b**). The capacity of each mAb to inhibit rosette formation or disrupt rosettes was studied as described in the “Methods” section. Assays were done on at least three distinct parasite cultures. A typical example is shown. The rosetting rate for each mAb concentration was recorded by microscopic examination and the results expressed as percent of rosettes observed in a test culture compared to a culture done in the absence of added mouse or rabbit reagent

Inhibition of rosette formation was assessed by cultivating synchronized ring stages for 24 h in the presence of a mAb and numerating the rosettes formed in presence of the mAbs at the end of this time period when the parasites reached the trophozoite-early schizont stage. Only mAbs reacting with DBL1 were able to efficiently prevent the formation of rosettes (Fig. 4a). D15-50, E20-76 and BD20E4 had similar rosette inhibition profiles and prevented 95–100 % rosette formation at a concentration of 1 μg mL^−1^. This high level of inhibition was observed with 10 μg mL^−1^ polyclonal rabbit IgG raised to bDBL1. D15-68 less efficiently prevented formation of VarO rosettes with low inhibition rates at 1 μg mL^−1^ and the least potent anti-DBL1 mAb was BDEE10. MAbs against the downstream domains failed to inhibit formation of rosettes at a concentration of 10 μg mL^−1^. Data are summarized in Table 2.

The capacity to disrupt VarO rosettes, i.e., break the interactions between the iRBC and the uninfected RBC, was evaluated by incubating monovariant cultures with the mAbs and numerating the iRBCs remaining engaged in rosettes after this incubation. This showed that the three mAbs that had similar inhibition profiles differed in their capacity to disrupt rosettes (Fig. 4b). BD20E4 was the most potent disrupter, with 50 % disruption at 0.7 μg mL^−1^ and 100 % disruption at 10 μg mL^−1^. MAb D15-50 was 100 % disruptive at 100 μg mL^−1^ concentration and only 60 % disruptive at 10 μg mL^−1^. MAb E20-76, on the other hand, was only 60 % disruptive at the highest concentration tested (100 μg mL^−1^). Surprisingly, BDEE10 displayed a much higher disruption capacity than D15-68. The other mAbs had no or quite marginal rosette-disrupting capacity (Fig. 4b; Table 2).

### Epitope specificity

**Fig. 5:**
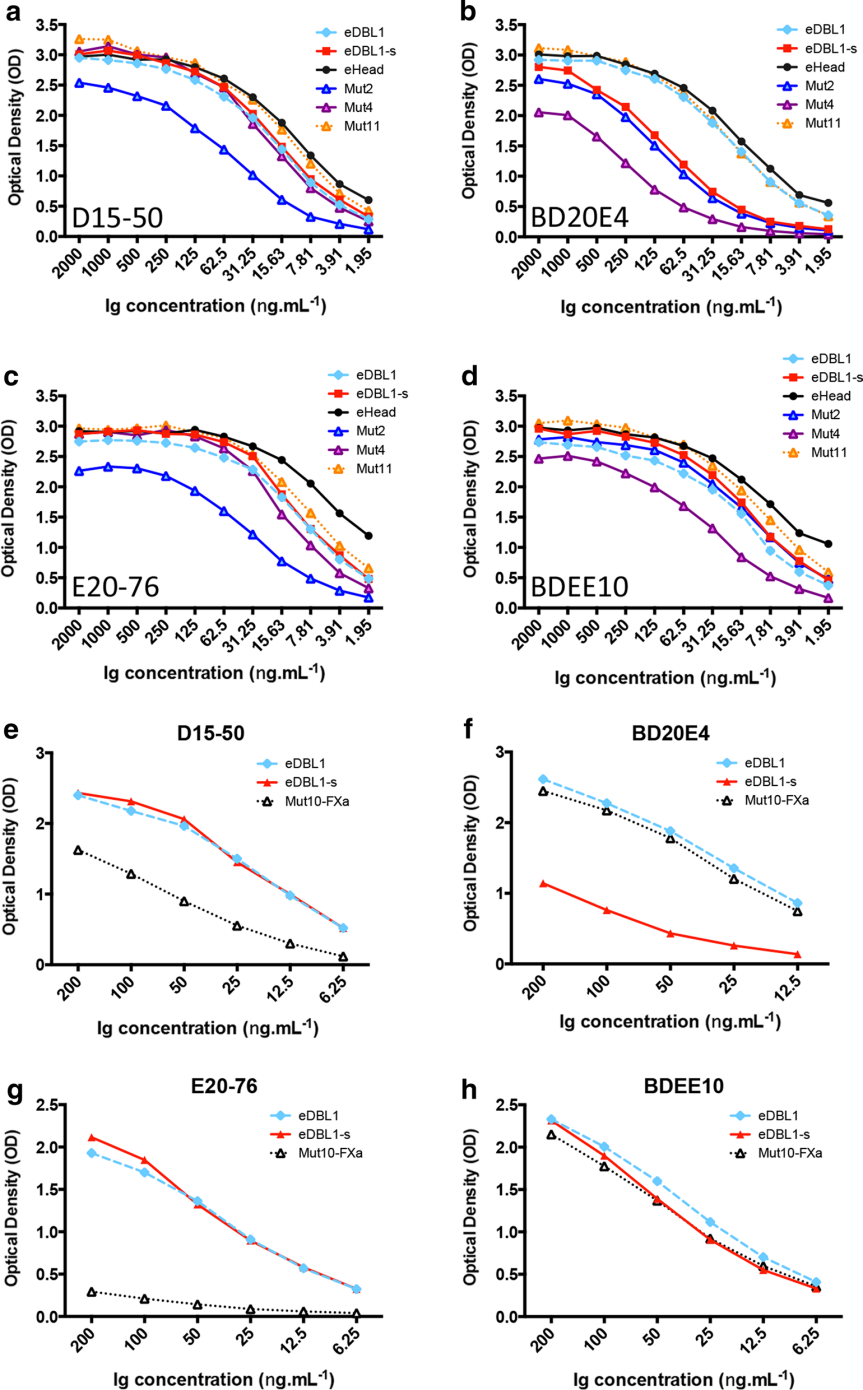
ELISA titration curves of individual mAbs on a panel of recombinant mutants of the DBL1 adhesion domain. **a**–**d** ELISA on eDBL1, eHead and Mut11 as well as mutants Mut2, Mut4 and parental eDBL1-s. **e**–**h** ELISA on eDBL1-s and Factor Xa-cleaved Mut10 and eDBL1. The following anti-DBL1 mAbs were used: D15-50 (**a**, **e**), BD20E4 (**b**, **f**), E20-76 (**c**, **g**) and BDEE10 (**d**, **h**)

To investigate epitope specificity of anti-DBL1 mAbs, reactivity of four mAbs (D15-50, E20-76, BD20E4, BDEE10) with a panel of recombinant eDBL1 proteins was analysed (Table 3). Previous work has shown that the eDBL1-s construct lacking the two C-terminal cysteine residues had essentially the same immunoreactivity with polyclonal sera raised to the longer bDBL1 or eDBL1 constructs [33]. This C-terminal deletion had limited impact on RBC binding [20, 28]. It however had a significant impact on binding of BD20E4. BD20E4 titres and reactivity were substantially lower on all constructs lacking 16 residues at the C-terminus and consequently the Cys19–Cys20 disulfide bond (Fig. 5b). Such a reduced reactivity with shorter constructs was not observed with the other mAbs (Fig. 5a, c, d; Table 3).

**Table 3:** Binding ratio of four anti DBL1-VarO mAbs on a set of recombinant domains with differing length and mutated residues

Ref	Protein	PfEMP1-VarO Residues (No Cys)	Mutations^a^	RBC binding^d^	Fold increase Kd heparin^e^	D15-50	E20-76	BD20E4	BDEE10	No tests
						Ratio to eDBL1^f^				
[27]	eDBL1	2–487 (20)	#	+++	ND	1	1	1	1	
[28]	eDBL1-s	2–471 (18)	# K87A	++	1	0.95	0.92	0.27	1.01	3
[22]	eDBL1-swt	1–471 (18)	Wild type	++++	ND	1	1	0.3	1	2
[22]	eHead	2–716 (31)	#	+++++	ND	1.4	1.5	1.3	1.8	2
						Ratio to parent eDBL1^f^				
[22]	Mut K87	2–487 (20)	# K87A	++++	ND	0.93	0.85	0.90	0.87	1
[22]	Mut10fXa^b^	2–487 (20)	# G66I Y67E V68G	Nil	ND	0.45	0.12	1.05	1.10	2
[22]	Mut11^c^	2–487 (20)	# G66I Y67E V68G	+++	ND	1.30	1.30	1.00	1.20	1
						Ratio to parent eDBL1-s^f^				
[28]	Mut1	2–471 (18)	# K87A K2A0 K32A K40A	++	115	0.66	0.67	0.79	0.76	2
[28]	Mut2	2–471 (18)	# K87A K95A K166A K179A	Nil	1	0.47	0.36	1.05	1.1	3
[28]	Mut3	2–471 (18)	# K87A K423A K424A K451A K456A	++	218	0.94	0.91	1.1	0.85	2
[28]	Mut4	2–471 (18)	# K87A K226A K227A K230A	Nil	11	0.94	0.84	0.42	0.45	3
[28]	Mut5	2–471 (18)	# K87A K404A K407A K410A	++	1	0.85	0.77	1.03	0.96	2
[22]	Mut8	2–471 (18)	# K87A R64A Y67A R69A	Nil	ND	0.87	1.40	0.85	0.97	1
[22]	Mut9	2–471 (18)	# K87A T88A Y90A E92A	+	ND	1.03	1.05	0.90	1.00	1

*ND* not determined

^a^Predicted N-glycosylation sites mutated (NxT/S to NxA)

^b^MBP-Mut10 was cleaved by Factor Xa located at the cloning site and at the internal site introduced at positions 66–69 (IEGR)

^c^DBL protein sequence identical to Mut10, but the cloning site (encoding a Factor Xa cleavage site) was changed for a thrombin cleavage site

^d^From ref [22]

^e^From ref [28]

^f^The mean ratio of reactivity was calculated as the mean of ratio to the parent protein observed for 3 successive dilutions in the linear part of the ELISA titration curves. The average ratio is shown for the proteins tested in 2-3 independent experiments

Reactivity with a set of recombinant proteins carrying specific mutations introduced into the eDBL1-s parent sequence was then analysed. Some of these mutations reduced heparin binding [28], while others abolished RBC binding, localizing the heparin-binding site and the RBC-binding site to opposite sides of the DBL1 domain [28]. Importantly, the various mutations have no major effect on conformation, as indicated by unaltered CD spectra [28, 20]. Although none of the mutations abolished reactivity of the set of mAbs tested, some did reduce binding as shown by reduced ELISA values and titres. The impact of specific mutations on mAb reactivity showed a clear partitioning (Fig. 5). D15-50 and E20-76 had a reduced binding to Mut2 (panels a and c, respectively), whereas binding of BD20E4 and BDEE10 to Mut4 was reduced (panels c and d, respectively). Cleavage of Mut10 by Factor Xa after residue R69 greatly reduced binding of D15-50 and E20-76 (panels e and g, respectively) but had no impact on binding of BD20E4 and BDEE10 (panels f and h, respectively). These data indicate that the mAbs bind to two adjacent DBL1 surface areas involved in RBC binding, since both the mutations harbored by Mut2 and Mut4 as well as Factor Xa cleavage after residue R64 were shown to abolish RBC binding [20]. There was no significant impact of the mutations of the heparin-binding site on mAb binding (Table 3).

Reactivity of the mAbs was further tested on a second panel of mutants derived from the eDBL1-s wild type sequence reported previously [20]. This included in particular specific mutations of the computationally derived ABO blood group binding site, introduced as single mutations (K95A) or double mutations (F145A K216A). None of these had any significant impact on the binding of the four mAbs (Additional file 3: Table S2, Figure S2).

Fine binding specificity differences could be observed within each mAb pair. The reduced binding of BD20E4 with the shorter constructs compared to the longer ones (eDBL1, Mut11, eHead) was not observed for BDEE10 and the impact of some mutations on binding differed between these mAbs (Fig. 5; Table 3). A trend for distinct mutant-specific reactivity was also observed for D15-50 and E2076. Binding of E20-76 to factor Xa-cleaved Mut10 was reduced compared to D15-50 (Fig. 5g, e respectively). Moreover, compared to D15-50, E20-76 displayed reduced binding to Mut11, Mut1, Mut2, Mut4, and Mut5, but enhanced binding to Mut8 (summarized in Table 3). In summary, binding profiles were specific for each of the two pairs identified by competition ELISA, and within each pair, specific for each mAb.

### Mapping of the binding site on the crystal structure of Palo Alto DBL1-VarO

**Fig. 6:**
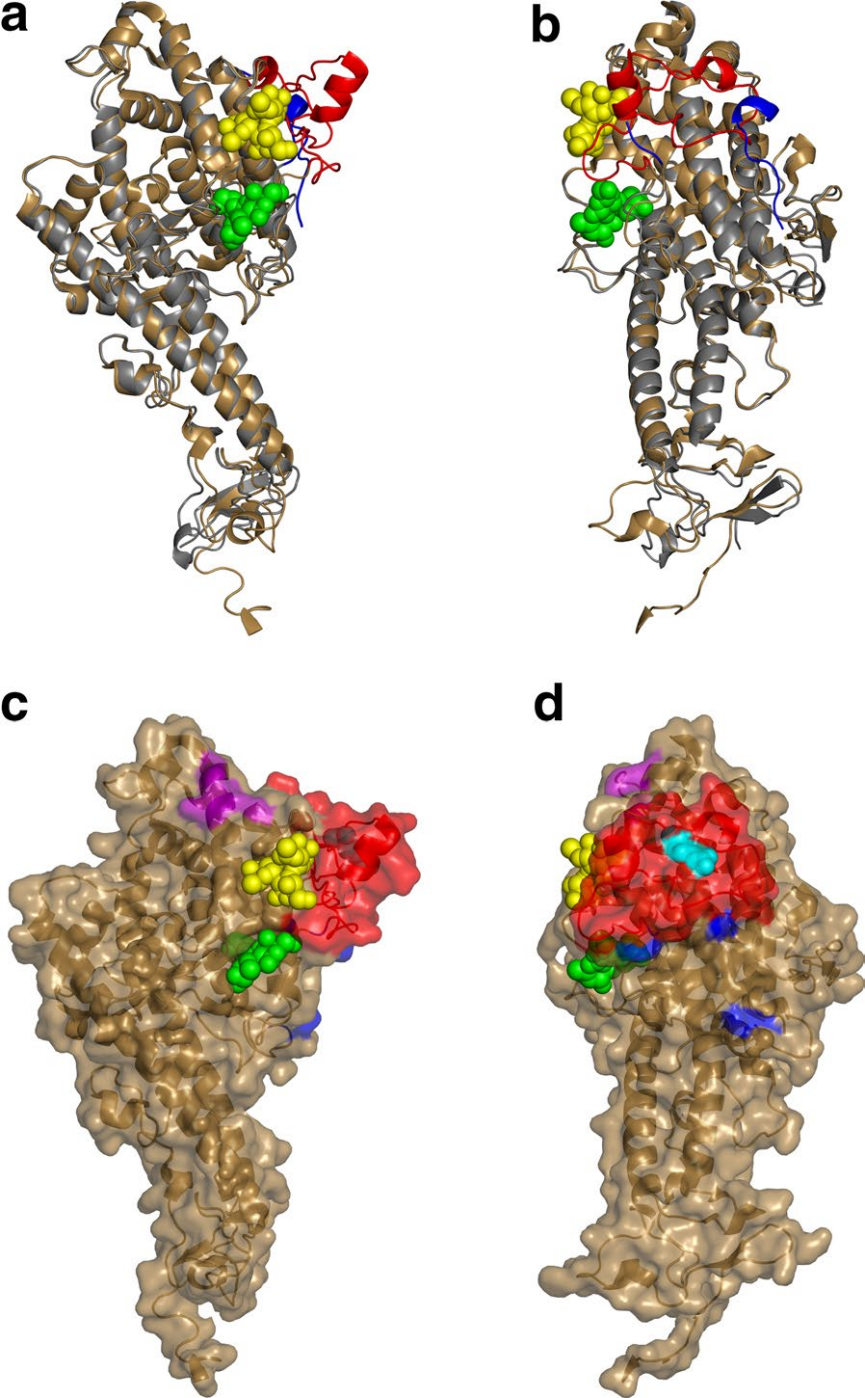
Localization of the mutations impairing binding of inhibitory mAbs on the DBL1 domain on the DBL1α1-VarO structure. **a**, **b** Superposition of the DBL1-VarO crystal structures from the intact and cleaved domains (PDB entry codes 2yk0 and 2xu0, respectively), shown in ribbon representation. The intact domain is shown in *brown* and the cleaved domain is shown in *grey*. The region undergoing the conformational change (from residues T51 to K95) is shown in *red* for the intact domain and in *blue* for the cleaved domain; only 16 of the 45 residues comprising this segment could be traced in the cleaved domain. This region forms one side of the binding sites for the blood group glycans that were predicted by docking calculations. The A and B blood group trisaccharides are shown as molecular surface representations in *yellow* and *green*, respectively. **a** is viewed from above the glycan binding site and **b** is rotated by 90° about a vertical axis. **c**, **d**. Positions of point mutations in the intact DBL1-VarO (PDB entry code 2yk0) that affect the binding of mAbs, showing the docked blood group trisaccharides, as in views (**a**, **b**). The structure is shown as a semi-transparent molecular surface with the ribbon representation. Mutations of the Mut2 protein are in *blue* and those of Mut4 are in *purple*. (Mutated residue K87, which is common to Mut2 and Mut4 is not shown as this does not affect the binding of either the mAbs or the blood group glycans; see text and Table 3). The region implicated in the conformational change upon cleavage is shown in *red* and the side chain of residue R69, shown as spherical atoms, is *cyan*. **c** is viewed from above the glycan binding site and **d** is rotated by 90° about a vertical axis

The structures of the DBL1-VarO domain (a DBL1α1.6 type) and the VarO Head protein (double domain DBL1α–CIDRγ) have been determined by X-ray crystallography (PDB entries 2xu0 and 2yk0 respectively) [20, 28]. The DBL1-VarO single domain, which was cleaved after residue R69 during expression and purification of the recombinant protein, did not bind to RBC [28]. The DBL1-VarO domain of the Head construct, by contrast, was produced intact and efficiently bound RBC [20]. Comparison of these two structures shows that the R69 cleavage leads to a significant conformational change in the polypeptide segment comprising the C-terminal region of NTS, the N-terminal region of subdomain 1 and the hinge connecting these two regions (in total, the segment between residues T51 and K95, inclusive) (Fig. 6a, b). Although this region could be completely traced in the electron density of the intact DBL1-VarO domain (PDB entry 2yk0), significant parts of this region were disordered in the cleaved protein and could not be built (PDB entry 2xu0). As this region is adjacent to the blood group binding sites on DBL1-VarO predicted by docking calculations, abrogation of RBC binding in the cleaved domain is most likely due to the conformational change (Fig. 6). Since the inhibitory and rosette-disrupting mAb D15-50 binds to the intact Head construct but poorly to the cleaved DBL1-VarO protein (See Table 3, Mut10fXa), this antibody is affected by the structural changes arising from the R69 cleavage. In addition, point mutations affecting the binding of D15-50 are displayed by Mut2 (K95A, K166A and K179A), which lie to one side of the predicted blood group A and blood group B binding sites, adjacent to the region that undergoes the conformational change induced by the R69 cleavage (Table 3; Fig. 6c, d). By contrast, BD20E4, a very effective rosette-disrupting mAb, binds less efficiently to Mut4, whose mutations (K226A, K227A and K230A) lie on the opposite side of the ABO blood group antigen binding site (Table 3; Fig. 6c, d). This is compatible with the observation that binding by BD20E4 is unaffected by the R69 cleavage and does not compete with the binding of D15-50. Although both Mut2 and Mut4 also carry the mutation K87A, the mutant Mut-K87, with only the K87A amino acid change, is fully recognized by these two mAbs (Table 3) and thus does not contribute to the epitope of either mAb. Of note, neither Mut2 nor Mut4 affected the binding of heparin, whose binding site on DBL1-VarO is predicted to be on the opposite side of the domain [28]. The ELISA results and structural epitope mapping thus present a coherent picture; the two potent rosette-disrupting mAbs, D15-50 and BD20E4, recognize two distinct epitopes adjacent to, but on opposite sides of, the predicted ABO blood group antigens-binding site.

## Discussion

This work investigated specificity and functionality of mAbs obtained from animals immunized with recombinant domains from PfEMP1-VarO. MAbs raised to four different PfEMP1-VarO domains were obtained; they reacted with the surface exposed PfEMP1-VarO protein and appear representative of the corresponding polyclonal sera with regard to potency of surface reactivity and inhibition of adhesion.

Potent mAbs were obtained against DBL1 that efficiently inhibited formation of rosettes down to quite low concentrations and efficiently reversed rosetting. This confirms previous data showing that polyclonal sera raised to the correctly folded, functional adhesion domain efficiently block and disrupt Palo Alto VarO rosetting [20, 33]. MAbs BD20E4, D15-50 and E20-76 were more potent inhibitors, and mAbs BD20E4 and D15-50 were more potent disrupters, than the polyclonal rabbit IgG raised against bDBL1 (Fig. 4). This is remarkable as VarO rosettes are large (called giant rosettes by others [38, 39]) and involve strong interactions that cannot be mechanically disrupted, in contrast to other rosetting parasites such as IT4R29 [23, 40]. As such, these mAbs represent precious tools to dissect key molecular interactions involved in rosetting. It is worth noting that design of the recombinant domain is important to the induction of potently blocking antibodies. Indeed, antibodies elicited against a PfEMP1-VarO DBL1 domain lacking a stretch of 89 C-terminal residues compared to DBL1 expressed here (and missing 5 out of 20 Cys residues, thereby preventing formation of the Cys324–Cys442, Cys354–Cys470, Cys377–Cys467 and Cys477–Cys483 disulfide bonds) only partially inhibited Palo Alto VarO rosetting [41].

Recently, rosetting was shown to occur in the absence of PfEMP1. In parasites in which PfEMP1 trafficking to the red cell membrane was impaired by disruption of the *Pfmahrp1* gene, variant STEVOR proteins were shown to mediate rosetting through binding to glycophorin C [42]. A-RIFINs were identified as a mediator of rosetting involving binding to blood group A antigens, when PfEMP1 was removed by trypsin treatment or blocked by adding PfEMP1-specific antibodies [41]. This led some authors to conclude that A-RIFINs “are conceivably the main ligand of this host-parasite interaction” and that it is possible that “PfEMP1 might work as an accessory or alternative interaction to reinforce by multiple contact points these protein-carbohydrate interactions that are usually weak” [43]. The potent rosette blocking and disruption capacity shown here for single mAbs reacting solely with PfEMP1 in Palo Alto 89F5 VarO extracts (Fig. 3) does not support such conclusions, but rather indicates that when PfEMP1 is expressed and surface-exposed, i.e., the normal condition of *P. falciparum* parasites, it is a major determinant of rosetting as PfEMP1-specific mAbs readily inhibit and disrupt rosettes.

Analysis of reactivity to a panel of constructs showed that some DBL1 mutations reduced but did not abolish binding of the mAbs, although they did completely abolish RBC binding of the recombinant domain [20]. MAbs interfering with rosetting bound to two distinct regions on the surface of the DBL1 domain (Fig. 6) in proximity to the blood group trisaccharide binding site, which has been localized by computer docking and site-directed mutagenesis to a restricted surface area situated at the interface of subdomain 1 and subdomain 2 in the vicinity of the NTS-DBL1α_1_ hinge region [20]. This is consistent with inhibition of rosetting by impairing access to the RBC-binding site. The close proximity or partial overlap of the BD20E4 epitope with the RBC-binding site is reminiscent of findings observed for the binding site of the 24E9 Fab mAb, which overlaps with the ICAM-1 binding site on the surface of the DBLβ3-D4 domain of PfEMP1-PFD1235w [44]. None of the anti-DBL1 VarO mAbs appear to bind to the region of subdomain 3 identified as the binding site of mAbs that potently disrupted FCR3S1.2 (alias IT4Var60) or R29 (alias IT4Var9) rosettes [45] located in a different region of the DBL1 surface (see Additional file 4: Figure S3). Interestingly, the RBC-binding site of the DBL1α-IT4var60 domain has recently been localized adjacent to the RBC-binding site of DBL1α-VarO [46]. Moreover, mapping of human antibody reactivity with short linear-peptides of DBL1α-FCR3S1.2 indicated the presence of epitopes recognized by anti-rosetting antibodies within subdomains 1 and 2 [47]. Although these data are consistent with the findings reported here, it is possible that other parts of the DBL1 domain contribute to optimal display of the binding site and as such, can be targeted by rosette disrupting antibodies. It is worth noting that both titres and OD values observed with BD20E4 decreased when the constructs lacked the C-terminal residues forming the hinge with CIDR, which are located far from the RBC-binding area [20]. The conservative conclusion from the work presented here is that mAbs displaying a potent rosette-inhibition and rosette-disruption activity bind with non-overlapping sites located close to the RBC-binding site area, but this does not exclude contribution of additional regions of the molecule or existence of additional important epitopes.

Most mAbs studied here targeted reduction-sensitive epitopes, including mAbs M21-17 and M21-30, which had an unusual profile as they recognized reduction-sensitive epitope(s) on both PfEMP1-VarO and the recombinant DBL1 domain but did not stain the VarO iRBC surface. The possibility that the binding sites are surface-exposed but masked by some serum component is ruled out by the lack of reactivity with the PfEMP1-VarO protein on immunoblots of Palo Alto 89F5 VarO SDS-extracts (Fig. 3). This indicates that the epitopes in question depend on the proper formation of disulfide bonds and the correct folding of the protein but are lost upon iRBC surface display.

Heparin and sulfated glycosaminoglycans are potent inhibitors of Palo Alto VarO rosetting [27] as well as of multiple rosetting types [24, 25, 46, 48–51]. This suggests the presence of some common motif/epitope shared by rosette-forming parasites, which constitutes an attractive intervention target. None of the mAbs isolated here proved sensitive to the presence of mutations that drastically reduced heparin binding [28]. Interestingly, two potent FCR3S1.2 rosette-disrupting mAbs also failed to map to the heparin-binding area of the expressed PfEMP1-IT4var60 molecule [46]. Although these properties prevent dissecting the molecular basis of VarO-heparin binding using the available inhibitory mAbs, it confirms that the heparin-binding site and the RBC-binding sites are located far from each other on the DBL1-VarO surface.

There was some relationship between ELISA titres and surface IFA titres and MFImax. The mAb with the lowest titre (G8-49) had the lowest surface reactivity, and interestingly, both ELISA titres and surface reactivity were lower than mAb N6-37, also a CIDR binder. In contrast, the mAbs with the highest ELISA titres (the anti-DBL2 mAbs B12-42 and B12-15 and the anti-DBL4 mAb D18-94) did not have the highest IFA titres and MFImax. This may reflect reduced accessibility of the individual domains on the iRBC surface compared to the N-terminal domains. Previous findings showed limited quantitative relationship between ELISA reactivity and surface staining with polyclonal sera [30, 33]. Interestingly however, there was a good relationship between MFImax and rosette inhibition. The two most potent disrupters, BD20E4 and D15-50 had the highest IFA titres and the highest MFImax, mAbs D15-68 and E20-76 had intermediate MFImax and rosette inhibition potency, while BDEE10, the least efficient inhibitor had the lowest MFImax. In contrast, there was poor relationship of MFImax and/or IFA titres with the rosette disruption potency (Table 2). The rosette-inhibition assay was more sensitive than rosette disruption, confirming data from other rosetting types [15]. However, there was no obvious relationship between the two assays. This is particularly striking for mAbs BD20E4, D15-50 and E20-76, which had similar rosette inhibition profiles and dissimilar rosette-disruption capacity. Whether this reflects difference in binding affinity for PfEMP1-VarO or different levels of steric hindrance within the cellular aggregates remains to be established. Affinity of the various mAbs for DBL1-VarO was not explored here, because of the unclear relevance of binding constants for the recombinant antigen with regard to binding to the iRBC surface-exposed PfEMP1-VarO. PfEMP1 is displayed as a multimodular protein (downstream modules may influence binding of inhibitory mAbs) and moreover by a specialized membrane structure, the knob, where it is presented at a high concentration. Moreover, rosette inhibition and rosette disruption involve impairment or displacement of interactions with long blood group saccharides expressed on a variety of RBC surface molecules and steric hindrance is an issue.

## Conclusions

The set of anti-VarO mAbs described here will facilitate future studies to design soluble rosetting inhibitors and dissect the specificity of human responses to the VarO antigenic variant, that is commonly recognized by humans living in malaria-endemic areas [13, 29, 30]. Importantly, all mAbs failed to react with the iRBC surface of related rosette-forming variants such as R29/IT4var9 or 3D7/PF13_0003, like polyclonal antibodies raised to the cognate DBL1 domains [30]. This confirms the variant-specific surface reactivity observed with a panel of mAbs to the R29/IT4var9 or FCR3S1.2/IT4var60 DBL1 proteins [45]. In line with this, none of the anti-VarO mAbs reacted with the region of subdomain 2 shown to induce variant-transcending antibodies [52], which is distant from the binding site areas of the anti-VarO mAbs and partially masked by the surface exposed NTS-VarO domain (see Additional file 4: Figure S3). The identification of a restricted surface area as the binding site of inhibitory mAbs opens the way for fine mapping of the variant-specific interactions of the inhibitory mAbs with the DBL1 domain. This information will be essential to better understand the specificity of antibodies elicited by infection in humans [13, 29, 30, 47] and determine which natural responses should be harnessed by vaccination and which additional specificities should be elicited by vaccination.

## Additional files

10.1186/s12936-015-1016-5 Impact of biotinylation of mAbs raised to bDBL1 on their ELISA reactivity. Dose-dependent reactivity of biotinylated mAbs assessed by ELISA on serial dilutions of bDBL1 antigen. ELISA plates with coated with decreasing concentrations of bDBL1 (threefold dilution series in PBS buffer), and processed as described in the “Methods” section. Pairs of biotinylated and native mAbs were tested in parallel at a dilution of 20 ng mL^−1^.
10.1186/s12936-015-1016-5 Summary table of competition ELISAs. Competition ELISA were carried out as described in section “Methods”. In brief, saturating concentrations of unlabelled monoclonal D15-50, D15-68, E20-76, BD20E4, BDEE10, M21-17 and M21-30 IgG were incubated with eDBL1-coated plates for 2 h at 37 ℃, unbound IgG were washed out and biotinylated D15-50, E20-76 or BD20E4 IgG were added to individual wells at a concentration previously determined to generate a signal of approximately 1 OD after incubation at 4 ℃ for 20 min. Binding of the biotin-labelled IgG was monitored using streptavidin-labelled horseradish peroxidase.
10.1186/s12936-015-1016-5 Titration curves of individual mAbs on a panel of recombinant mutants of the eDBL1-s or eDBL1-s-wt adhesion domains. The list of mutants analysed and their characteristics are given in the Table included. The ELISAs for D15-50 (A) E20-76 (B), BD20E4 (C) and BDEE10 (D) are each split into four panels for clarity. Recombinant proteins were produced as described [20, 28] and used to coat ELISA plates as described in section “Methods”. Pure monoclonal IgG were used for the ELISAs.
10.1186/s12936-015-1016-5 Further views of the DBL1 structure showing the localization of the mutated residues and other putative binding sites (**a**, **b**) The DBL1-VarO structure is shown as a molecular surface in two views that are rotated by 180° with respect to each other. The color scheme is as in Fig. 6: the main body of the DBL1-VarO domain is shown in *brown*, with the changed Mut2 residues in *blue*, the Mut4 residues in *purple*, residue R69 in *cyan* and the region undergoing conformational change after cleavage at R69 in *red*. The Blood Group A and B trisaccharides are shown as spherical atoms in *yellow* and *green*, respectively. The region of the subdomain 3 identified by Angeletti et al. [45] as the binding site of rosette-inhibitory antibodies is shown in *orange*. The subdomain 2 region containing the R E D W W T I N R E Q I W K A sequence (*magenta*), i.e., the VarO orthologue of the **R** E Y/**D** **W** **W** A/**T** L/**I** **N R** K/D E/**Q**/D V **W** **K** **A** (identical amino acids in *bold*) sequence containing the ALNRKE sequence motif described by Blomquist et al. [52] as inducing strain-transcending antibodies that react with the iRBC surface is shown in *pale blue*. This segment is partially buried by the N-terminal region of the domain called NTS.
