# Supplementary figures and images for: Functional analysis of monoclonal antibodies against the Plasmodium falciparum PfEMP1-VarO adhesin
Source: Malar J. 2016 Jan 15;15:28. doi: 10.1186/s12936-015-1016-5 (PMC4715314; doi:10.1186/s12936-015-1016-5)

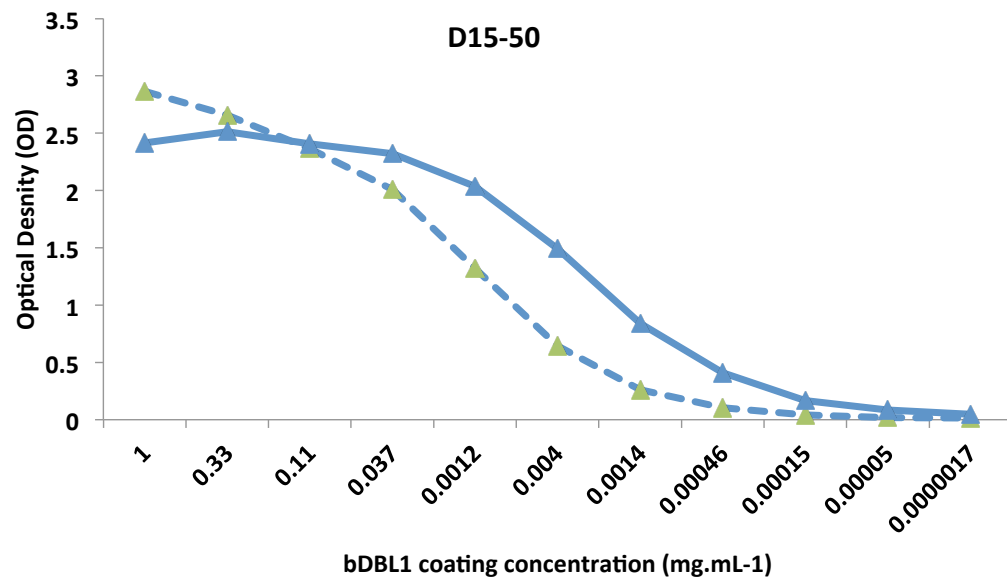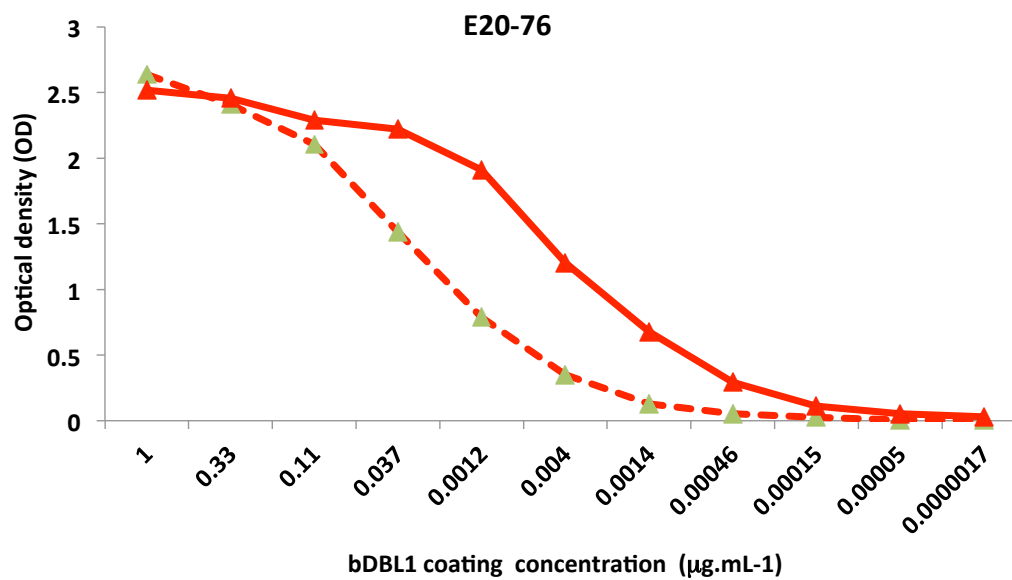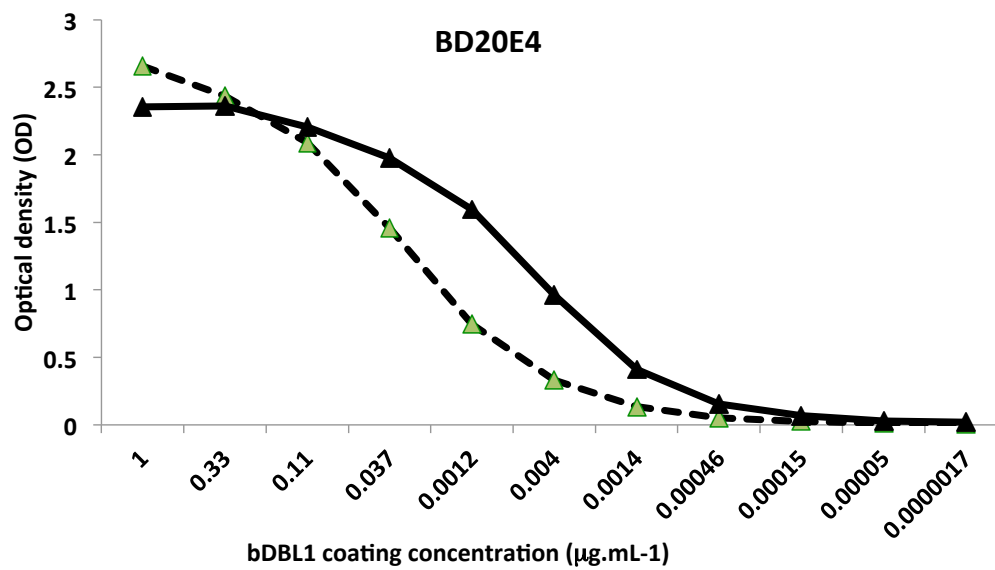

Supplement: Supplementary file 1 — 10.1186/s12936-015-1016-5 Impact of biotinylation of mAbs raised to bDBL1 on their ELISA reactivity. Dose-dependent reactivity of biotinylated mAbs assessed by ELISA on serial dilutions of bDBL1 antigen. ELISA plates with coated with decreasing concentrations of bDBL1 (threefold dilution series in PBS buffer), and processed as described in the “Methods” section. Pairs of biotinylated and native mAbs were tested in parallel at a dilution of 20 ng mL−1. [file 12936_2015_1016_MOESM1_ESM.pdf]

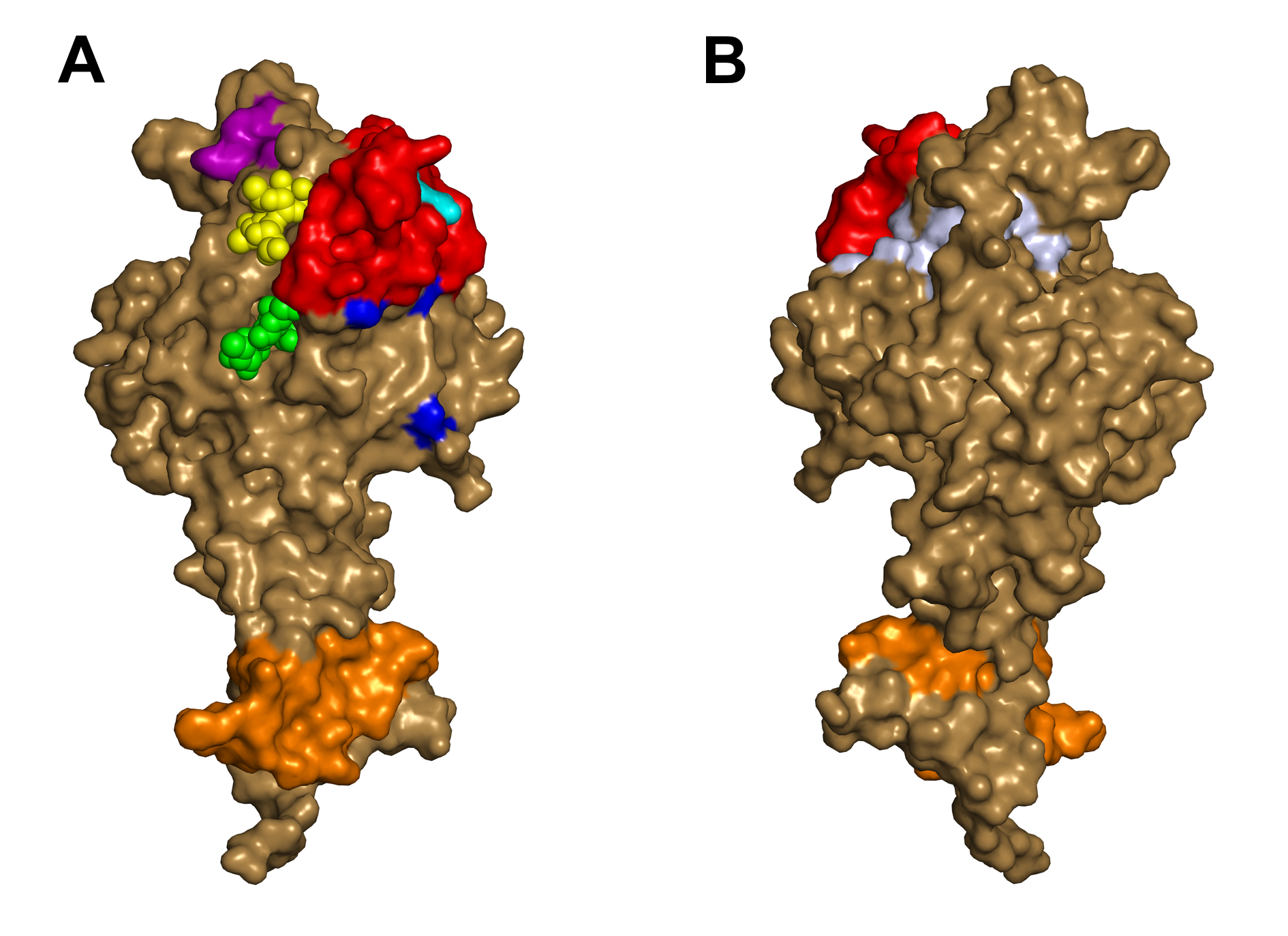

Supplement: Supplementary file 4 — 10.1186/s12936-015-1016-5 Further views of the DBL1 structure showing the localization of the mutated residues and other putative binding sites (a, b) The DBL1-VarO structure is shown as a molecular surface in two views that are rotated by 180° with respect to each other. The color scheme is as in Fig. 6: the main body of the DBL1-VarO domain is shown in brown, with the changed Mut2 residues in blue, the Mut4 residues in purple, residue R69 in cyan and the region undergoing conformational change after cleavage at R69 in red. The Blood Group A and B trisaccharides are shown as spherical atoms in yellow and green, respectively. The region of the subdomain 3 identified by Angeletti et al. [45] as the binding site of rosette-inhibitory antibodies is shown in orange. The subdomain 2 region containing the R E D W W T I N R E Q I W K A sequence (magenta), i.e., the VarO orthologue of the R E Y/D W W A/T L/I N R K/D E/Q/D V W K A (identical amino acids in bold) sequence containing the ALNRKE sequence motif described by Blomquist et al. [52] as inducing strain-transcending antibodies that react with the iRBC surface is shown in pale blue. This segment is partially buried by the N-terminal region of the domain called NTS. [file 12936_2015_1016_MOESM4_ESM.png]
