# Supplementary material for: Functional analysis of monoclonal antibodies against the Plasmodium falciparum PfEMP1-VarO adhesin
Source: Malar J. 2016 Jan 15;15:28. doi: 10.1186/s12936-015-1016-5 (PMC4715314; doi:10.1186/s12936-015-1016-5)
Supplement: Supplementary file 2 — 10.1186/s12936-015-1016-5 Summary table of competition ELISAs. Competition ELISA were carried out as described in section “Methods”. In brief, saturating concentrations of unlabelled monoclonal D15-50, D15-68, E20-76, BD20E4, BDEE10, M21-17 and M21-30 IgG were incubated with eDBL1-coated plates for 2 h at 37 ℃, unbound IgG were washed out and biotinylated D15-50, E20-76 or BD20E4 IgG were added to individual wells at a concentration previously determined to generate a signal of approximately 1 OD after incubation at 4 ℃ for 20 min. Binding of the biotin-labelled IgG was monitored using streptavidin-labelled horseradish peroxidase. [file 12936_2015_1016_MOESM2_ESM.pdf]

Biotinylated mAb	Competing mAb						
	D15-50	D15-68	E20-76	BD20E4	BDEE10	M21-30	M21-17
D15-50		30		10	10	0	0
E20-76		50		0	0	0	0
BD20E4	0	0	0			0	0
