# Supplementary material for: Functional analysis of monoclonal antibodies against the Plasmodium falciparum PfEMP1-VarO adhesin
Source: Malar J. 2016 Jan 15;15:28. doi: 10.1186/s12936-015-1016-5 (PMC4715314; doi:10.1186/s12936-015-1016-5)
Supplement: Supplementary file 3 — 10.1186/s12936-015-1016-5 Titration curves of individual mAbs on a panel of recombinant mutants of the eDBL1-s or eDBL1-s-wt adhesion domains. The list of mutants analysed and their characteristics are given in the Table included. The ELISAs for D15-50 (A) E20-76 (B), BD20E4 (C) and BDEE10 (D) are each split into four panels for clarity. Recombinant proteins were produced as described [20, 28] and used to coat ELISA plates as described in section “Methods”. Pure monoclonal IgG were used for the ELISAs. [file 12936_2015_1016_MOESM3_ESM.pdf]

Ref	Protein	PfEMP1-VarO Residues (No Cys)	Mutations[#]	RBC binding	D15-50	E20-76	BD20E4	BDEE10	No testsELISA with mAbs
					ratio to parent eDBL1-s				
[28]	eDBL1-s	2 - 471 (18)	# K87A	++					5
[28]	Mut5	2 - 471 (18)	# K87A K404A K407A K410A	++					3
[20]	Mut8	2 - 471 (18)	# K87A R64A Y67A R69A	Nil					1
[20]	Mut9	2 - 471 (18)	# K87A T88A Y90A E92A	+					1
					ratio to parent eDBL1-swt				
[20]	eDBL1-swt	1 - 471 (18)	Wild type	++++					4
[20]	Mut14	1 - 471 (18)	R64A	++					1
[20]	Mut15	1 - 471 (18)	T88A	+					1
[20]	Mut16	1 - 471 (18)	K87A	++					1
[20]	Mut17	1 - 471 (18)	K95A	Nil					1
[20]	Mut18	1 - 471 (18)	F145A K216A	+					2
[20]	Mut19	1 - 471 (18)	D147A K149A	Nil					1

N-glycosylation sites mutated (NxT/S to NxA)

Nil: no RBC binding

¶ from ref 20

	Ratio >1.5		Ratio >1.2 – 1.5		Ratio: 0.8 -<1.2		Ratio: 0.6 -<0.8

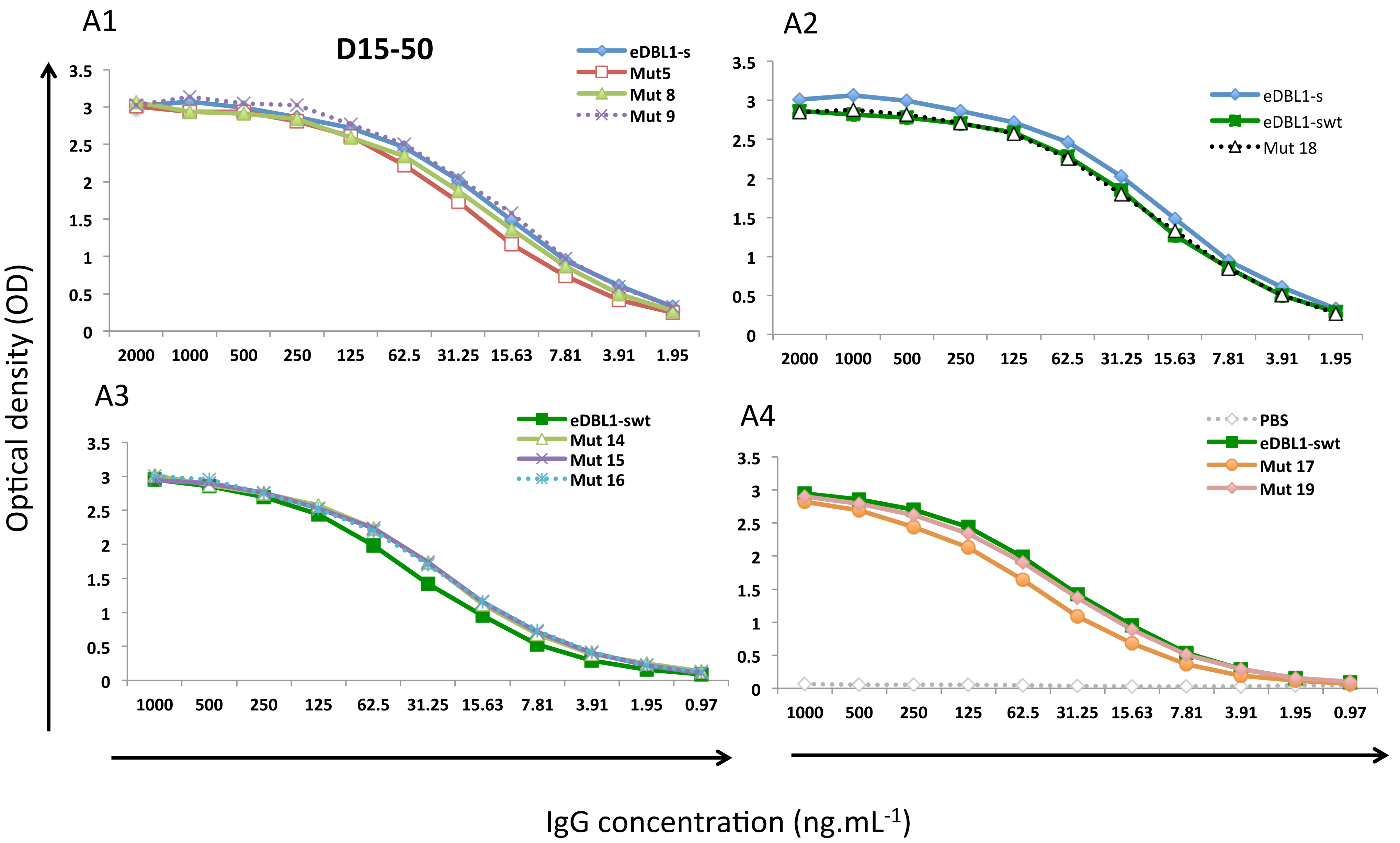
A1
D15-50
eDBL1-s
Mut5
Mut 8
Mut 9
A2
eDBL1-s
eDBL1-swt
Mut 18
A3
eDBL1-swt
Mut 14
Mut 15
Mut 16
A4
PBS
eDBL1-swt
Mut 17
Mut 19
Optical density (OD)
IgG concentration (ng.mL-1)

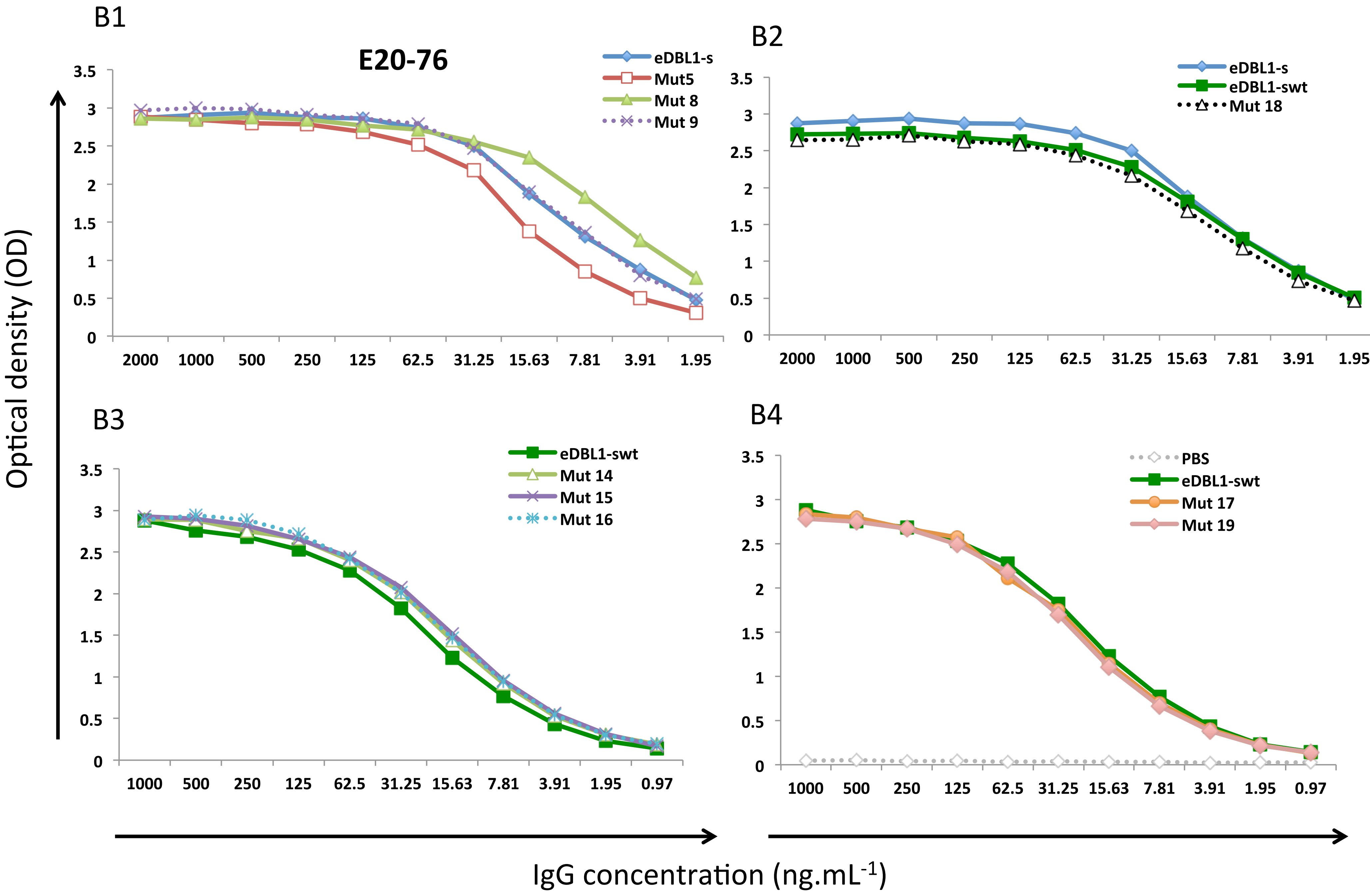

B1
E20-76
eDBL1-s
Mut5
Mut 8
Mut 9
3.5
3
2.5
2
1.5
1
0.5
0
2000 1000 500 250 125 62.5 31.25 15.63 7.81 3.91 1.95
B2
eDBL1-s
eDBL1-swt
Mut 18
3.5
3
2.5
2
1.5
1
0.5
0
2000 1000 500 250 125 62.5 31.25 15.63 7.81 3.91 1.95
B3
eDBL1-swt
Mut 14
Mut 15
Mut 16
3.5
3
2.5
2
1.5
1
0.5
0
1000 500 250 125 62.5 31.25 15.63 7.81 3.91 1.95 0.97
B4
PBS
eDBL1-swt
Mut 17
Mut 19
3.5
3
2.5
2
1.5
1
0.5
0
1000 500 250 125 62.5 31.25 15.63 7.81 3.91 1.95 0.97
Optical density (OD)
IgG concentration (ng.mL-1)

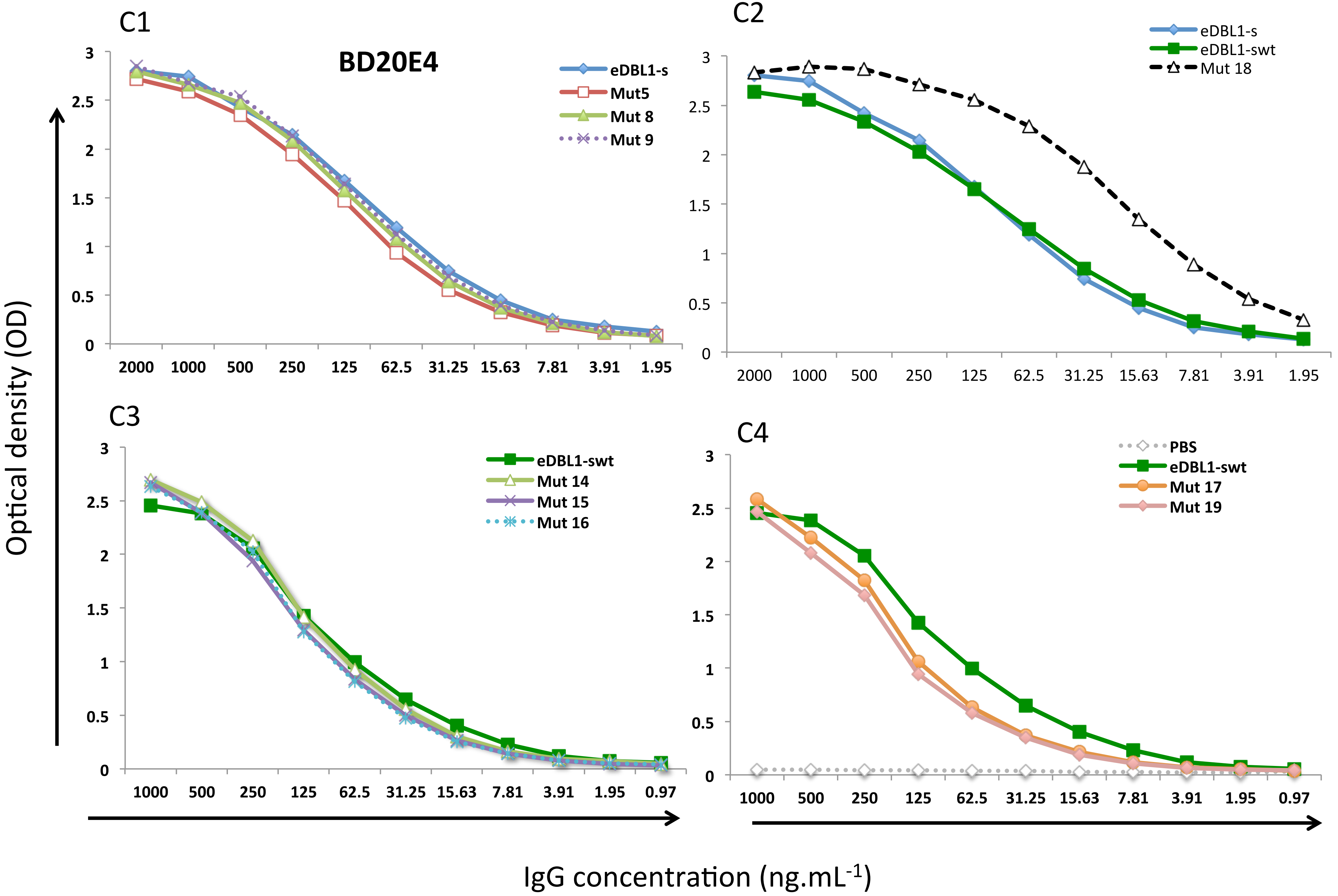

C1
BD20E4
eDBL1-s
Mut5
Mut 8
Mut 9
C2
eDBL1-s
eDBL1-swt
Mut 18
C3
eDBL1-swt
Mut 14
Mut 15
Mut 16
C4
PBS
eDBL1-swt
Mut 17
Mut 19
Optical density (OD)
IgG concentration (ng.mL-1)

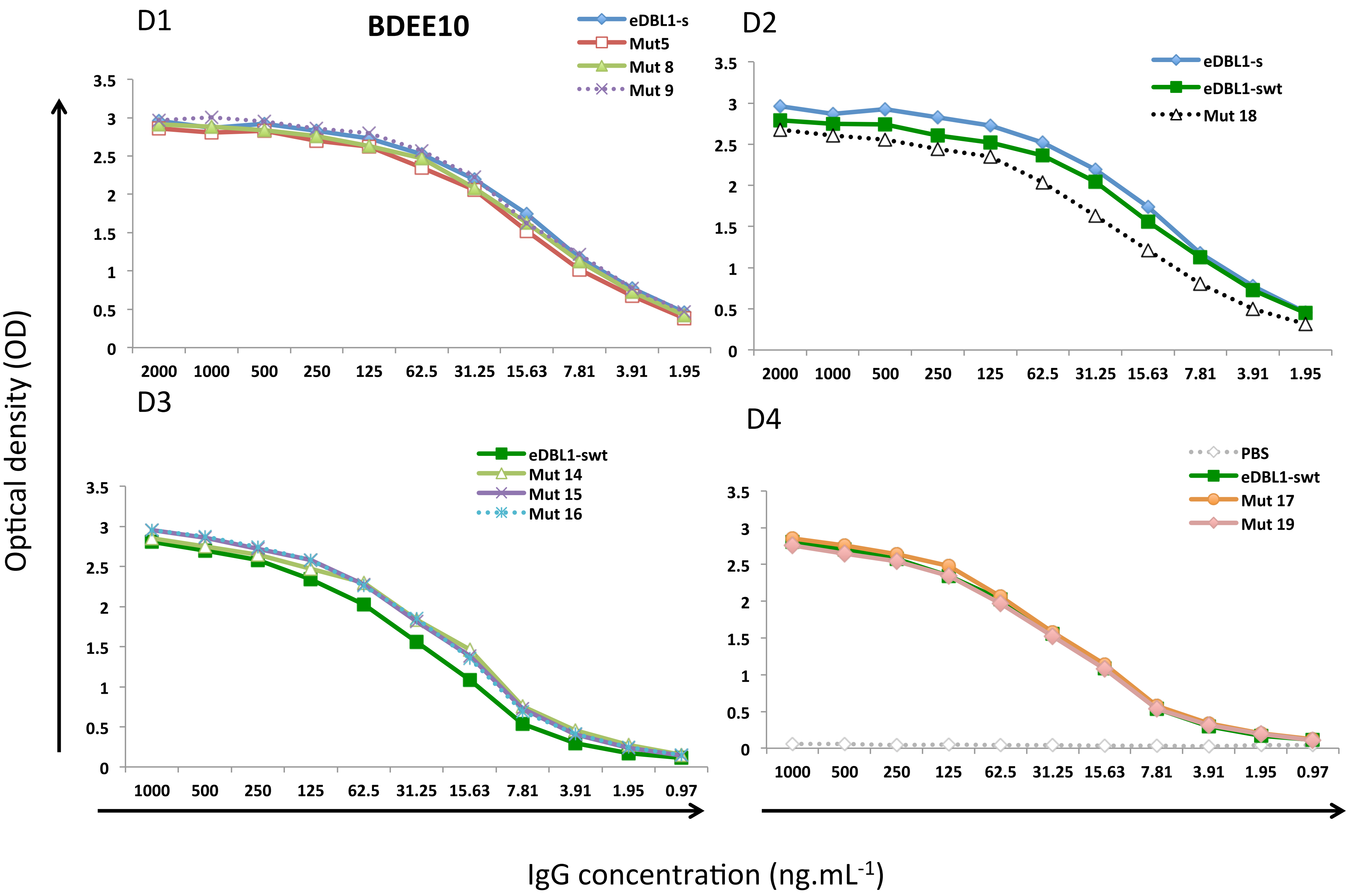
D1
BDEE10
eDBL1-s
Mut5
Mut 8
Mut 9
D2
eDBL1-s
eDBL1-swt
Mut 18
D3
eDBL1-swt
Mut 14
Mut 15
Mut 16
D4
PBS
eDBL1-swt
Mut 17
Mut 19
Optical density (OD)
IgG concentration (ng.mL-1)
2000 1000 500 250 125 62.5 31.25 15.63 7.81 3.91 1.95
1000 500 250 125 62.5 31.25 15.63 7.81 3.91 1.95 0.97
